# Factors affecting return to work in breast cancer survivors in Korea: a cross-sectional study

**DOI:** 10.4069/whn.2024.12.10

**Published:** 2024-12-30

**Authors:** Kate J. Sohn, Sung Hae Kim, Hyojin Lee, Sue Kim

**Affiliations:** 1 Yonsei University, Seoul, Korea; 2 College of Nursing, Mo-Im Kim Nursing Research Institute, Yonsei University, Seoul, Korea; 3 Department of Nursing, College of Health, Welfare and Education, Tongmyong University, Busan, Korea

**Keywords:** Breast neoplasms, Cancer survivors, Fatigue, Psychosocial functioning, Return to work

## Abstract

**Purpose:**

Return to work (RTW) has been understudied in Asian women with cancer, despite the increasing number of breast cancer survivors (BCS). This study examined RTW among Korean BCS, exploring its associations with cancer-related fatigue, quality of sleep, mental adjustment, and psychosocial factors.

**Methods:**

This cross-sectional study recruited BCS from a hospital, a breast cancer support group, and an online community in Korea between July and August 2019. We collected data on levels of fatigue, fatigability, quality of sleep, mental adjustment, and quality of working life. The analysis included data from 135 respondents who were employed prior to their cancer diagnosis. Descriptive statistics and multiple logistic regression analyses were conducted.

**Results:**

Although all participants were employed prior to diagnosis, only 57% remained employed afterward. Participants who returned to work reported significant levels of subjective fatigue (102.48±39.84), physical fatigability (28.14±11.34), borderline poor sleep quality (8.57±4.11), anxious preoccupation (23.33±4.54), and low satisfaction with quality of working life (39.68±21.51). Marital status (odds ratio [OR], 3.34; *p*=.027), time since breast cancer diagnosis (OR, 2.85; *p*=.028), anxious preoccupation (OR, 0.89; *p*=.021), and quality of working life (OR, 1.04; *p*=.010) were found to be predictors of RTW, explaining 34% of the variance.

**Conclusion:**

It is critical to address RTW-related difficulties in Korean BCS, and future RTW interventions should target cancer-related fatigue, anxious preoccupation, and quality of working life. Physical and psychosocial support is essential for BCS and their successful RTW.

## Introduction

As early detection and treatment improve, increasingly many breast cancer patients in Korea are being diagnosed at younger ages [1]. This often coincides with the prime child-bearing and working stages of life [2]. Substantial evidence indicates that cancer survivors are more likely to be unemployed than the general population [2], making the decision to return to work (RTW) and its management a significant concern in survivorship. RTW affects cancer survivors not only in terms of physical and mental health but also impacts their financial burden, quality of life (QoL), and more. Additionally, these impacts vary according to different healthcare systems, cultural models, and socioeconomic factors [2-4]. Research on the RTW of breast cancer survivors (BCS) in Korea is relatively new. Cultural differences specific to the RTW of Asian cancer survivors have been noted, such as the influence of Confucian values, higher levels of self-stigma, and self-consciousness about disclosing a cancer diagnosis [2]. More research is needed on the RTW experiences and challenges of non-Western populations in order to enable the development of more effective and inclusive RTW and overall breast cancer survivorship programs and resources [2-3,5].

Cancer-related fatigue (CRF) occurs in 60% to 90% of cancer survivors during treatment [6]. CRF does not resolve immediately after treatment ends and is challenging to diagnose and manage, leading many survivors to experience ongoing fatigue even after recovery [7,8]. Additionally, posttreatment CRF is often more complex due to its potential association with long-term psychological adverse events, including hormonal changes, pain, sleep disturbances, anxiety, and depression [6-7,9]. Furthermore, CRF has been found to be negatively associated with RTW during breast cancer treatment and is linked to cognitive impairments, work limitations, and work burdens [10]. Persistent CRF can significantly hinder the ability of BCS to return to and maintain employment, thereby affecting their QoL, financial stability, and social reintegration [7,11]. This ongoing fatigue underscores the need for tailored nursing approaches that provide both physical support and psychosocial interventions to manage fatigue effectively [12]. However, research on CRF among Korean BCS, particularly concerning RTW, remains insufficient. A recent study explored the fatigue-depression-anxiety symptom cluster within a Korean BCS cohort, finding that increased fatigue correlates with greater psychological distress [13]. While existing research on similar symptom clusters has demonstrated a negative impact on QoL, many studies have not simultaneously addressed critical factors such as sleep quality and work-related challenges [14]. Additionally, mental health factors and coping skills are critical concerns that should be included in both research and subsequent interventions [15,16].

Therefore, this study aimed to identify the impact of fatigue, sleep quality, physical activity, ability to adjust to cancer, depression, cognitive function, and quality of working life on the RTW of Korean BCS. It also examined the sociodemographic and clinical context influencing these factors.

## Methods

**Ethics statement:** Ethical approval for the study was granted by the Ethics Committee of Severance Hospital (No. 4-2019-0557). Eligibility of all participants was confirmed, and they were provided with detailed study information. Voluntary informed consent was obtained from each participant before administering the questionnaire.

### Study design

This cross-sectional study utilized descriptive a correlational research design and adhered to the STROBE guidelines (https://www.strobe-statement.org/).

### Sample and sampling

**Figure 1.**
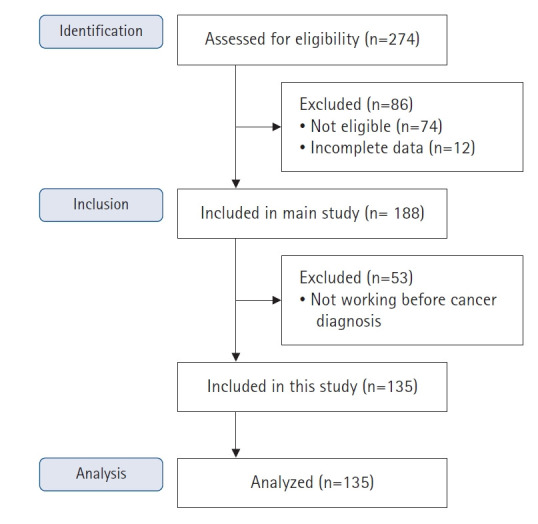
Flow diagram of recruitment.

Korean BCS were recruited at the breast sonography center of Severance Hospital, Yonsei University Health Systems in Seoul, Korea, as well as from a breast cancer support group and an online cancer community through convenience sampling from July 29 to August 31, 2019. Referring to a review study of intervention programs for RTW among cancer survivors [17], which included participants aged 18 to 75 years, and considering years of active life, this study included participants aged 20 to 70 years. Eligible participants were women diagnosed with breast cancer within the past 10 years, aged between 20 and 70 years. BCS with underlying diseases that could affect fatigue (e.g., cardiopulmonary or thyroid disease), diagnosed psychiatric conditions (e.g., panic disorders or schizophrenia), cancer recurrence, or additional cancers were excluded. The sample size was calculated using the G*Power ver. 3.1.9.7 program [17] for logistic regression analysis, with a significance level of α=.05, an odds ratio (OR) of 2.0, and a power of 0.80 [18]. The total required sample size was 138. Of the 200 participants recruited, those who missed more than five questions or did not complete the survey were considered to have provided incomplete responses (n=12). From the remaining 188 respondents, data from 53 participants who were not employed prior to their cancer diagnosis were excluded from the analysis. Thus, a total of 135 BCS who were employed before their cancer diagnosis were analyzed in this study. Figure 1 outlines the process of enrollment and data collection.

### Study variables and measures

Participants completed a self-reported questionnaire, which was available either online or in paper form, according to their preference. Permission for use was obtained from the original developers and/or the authors of the translated versions.

#### Cancer-related fatigue

Fatigue was measured in terms of subjective fatigue and activity-related fatigue (fatigability). The Korean version of the Revised Piper Fatigue Scale (K-R-PFS) [19], consisting of 19 items, was used to measure subjective fatigue in four subdomains: behavioral/severity (six items), affective meaning (four items), sensory (four items), and cognitive/mood (five items). On an 11-point Likert scale (0–10) higher summed scores (possible range, 0–190) indicate greater fatigue. The K-R-PFS is a reliable and valid measure (Cronbach’s α =.84–.93) [19], and Cronbach’s α was .97 in this study. For activity-related fatigue, the Pittsburgh Fatigability Scale [20] Korean version (K-PFS) [21] was used. The 10 items measure fatigue based on the intensity and duration of common activities by classifying areas of activity according to the required energy levels. On a 6-point Likert scale (0–5), higher summed scores for physical and mental fatigability (possible range, 0–50 each) indicate more severe fatigue. The internal consistency was good at the time of development (Cronbach’s α=.86) [20] and excellent in this study (Cronbach’s α for the physical domain=.911, Cronbach’s α for the mental domain=.911, and total Cronbach’s α=.948).

#### Quality of sleep

The Pittsburgh Sleep Quality Index [22] Korean version (PSQI-K) [23] was used to measure sleep quality in the past month, with 19 items covering seven subdomains (subjective sleep quality, sleep latency, duration of sleep, habitual efficiency, disturbances, use of sleeping medication, and dysfunction during the day) [22]. Scored from 0 to 3, higher global scores (possible range, 0–21) are correlated with worse sleep quality and scores >8.5 indicate a sleep disorder in clinical samples [23]. Internal consistency was good (Cronbach’s α=.83) at development [22] and for the PSQI-K (Cronbach’s α=.84) [23], and adequate in this study (Cronbach’s α=.72).

#### Mental adjustment to cancer

The Mini-Mental Adjustment to Cancer scale [24] in the Korean version (K-Mini-MAC) [25] is a 29-item tool that evaluates helplessness-hopelessness (eight items), anxious preoccupation (eight items), fighting spirit (four items), cognitive avoidance (four items), and fatalism (five items), on a 4-point Likert scale (1–4). Summed scores are calculated for each subdomain, and higher scores indicate stronger adaptive responses or perceptions of control. The K-Mini-MAC was found to be reliable, valid, and culturally acceptable for the Korean cancer population [25]. The subscales in this study also demonstrated acceptable internal consistency (Cronbach’s α=.62–.92).

#### Quality of working life

The 23-item Quality of Working Life Questionnaire for Cancer Survivors (QWLQ-CS) [26] Korean version [27] measured QoL in work capacity across five subdomains: meaning of work (four items), perception of job situation (five items), work environment/atmosphere (five items), understanding and recognition in the workplace (five items), and problems due to health issues (four items). A 6-point Likert scale (1–6) is used to calculate standardized scores (0–100) [26]. Higher standardized scores indicate better quality of working life. Participants were instructed to reply to the QWLQ-CS based on their latest work experience if they intended to work again within 1 year. Internal consistency was established in the original study (Cronbach’s α=.89) [26] and in a Korean sample (Cronbach’s α=.91) [27] and was also high in this study (Cronbach’s α=.97).

#### Participant characteristics

Sociodemographics, disease-related characteristics, various symptom experiences, and work-related characteristics were obtained from all participants.

### Procedures

The study was conducted from July 29 to August 31, 2019. We posted a participant recruitment notice in the clinic and on an online cancer community platform, allowing individuals to voluntarily access the online survey, which included an information sheet and a consent form. Additionally, the study was promoted during a BCS support group event, where trained research assistants obtained consent from interested participants. To prevent duplicate responses in both the online and offline formats, each participant was assigned a unique identification code. Before taking the survey, respondents were asked if they had previously participated in this study. For those completing paper questionnaires, we collected the filled-out forms in a box at the conclusion of the event. A small gift (approximately 5 US dollars) was provided to all participants. We also provided contact information for supportive resources, including a mental health care hotline and breast cancer support group details, for all participants’ convenience.

### Data analysis

Descriptive statistics were employed to analyze the demographic characteristics of participants and other variables. Correlation analyses, the independent t-test, the chi-square test, and the analysis of variance were used to identify differing factors. Multiple logistic regression analysis was performed to identify relevant predictors of RTW. All statistical analyses were conducted using IBM SPSS ver. 25 (IBM Corp., Armonk, NY, USA). Statistical significance was established at *p*<.05.

## Results

### Participant characteristics

Table 1 displays the characteristics of the 135 participants. The average age was 44.72±9.09 years. A majority of the participants were married, including those who were divorced, separated, or widowed (78.5%), and had children (67.4%). Most of the BCS had at least a college education (71.1%), with 43% reporting a monthly income between 3 million and 5 million Korean won (approximately 2,680–4,474 US dollars, which is considered to reflect a middle-class income, considering the 2019 national household average monthly income of 4.77 million Korean won [28]). About 45.9% of participants reported their economic burden as ranging from a little to very burdensome. Most participants had been diagnosed within the past 5 years (94.8%) and were at stages 0 to 2 of their condition (86.7%). A large majority had undergone breast surgery (87.4%), and 24.5% were also undergoing treatment. While 98.5% experienced pain in the week prior to taking the survey, the majority described their pain as mild and did not require painkillers (65.9%). Approximately one-fourth (24.4%) reported having an underlying disease, such as cardiovascular, pulmonary, or musculoskeletal disease. Despite 74.8% of participants being under 50 years of age, 60.7% reported experiencing menopausal symptoms and discomfort. A significant majority were unable to recall ever receiving fatigue-related education (91.9%), and those who did receive such education reported it lasted about an hour.

Seventy-seven participants (57.0%) reported that they continued working after their diagnosis. The primary reasons for changes in work status were personal physical issues (71.9%), including changes in appearance or body, fatigue, fitness, and therapy side effects. This was followed by personal psychological reasons (63.0%), such as depression, anxiety, worsening attention or memory, and altered perceptions of work meaning. Workplace-related reasons (44.4%) included the nature of work not accommodating concurrent work and treatments, conflicts with supervisors or colleagues, an organizational culture that fails to understand BCS, lack of company policies or systems to support BCS, prejudice and discrimination at work, and challenges in managing personal business. Lastly, reasons connected to family and neighbors (35.6%) involved disruptions in family life and difficulties in balancing daily work with household responsibilities.

### Characteristics of the main variables

Results for the main variables are presented in Table 2. Participants reported high levels of fatigue, with mean levels of subjective fatigue at 94.88±37.47, physical fatigability at 25.73±10.56, and mental fatigability at 21.87±11.15. The quality of sleep was borderline poor, as indicated by a total mean score of 7.81±3.85. Mean levels for the adjustment to cancer subsections were moderate: helplessness-hopelessness at 15.16±4.80, anxious preoccupation at 21.81±4.60, fighting spirit at 11.78±1.97, cognitive avoidance at 10.56±2.59, and fatalism at 14.31±2.59. The mean score for quality of working life (48.95±20.28) indicated a poor level.

### Differences in return to work according to general characteristics

More single participants successfully returned to work compared to those who were married or had been married (*χ*^2^=5.34, *p*=.021). It was more common for participants to return to work between 2 to 5 years after diagnosis than within the first 2 years after diagnosis (*χ*^2^=8.05, *p*=.018). Participants who had completed their chemotherapy, radiation therapy, or targeted therapy, as well as those who had not received any treatment, experienced a higher frequency of RTW compared to those currently undergoing treatment (*χ*^2^=10.55, *p*=.004). No statistical significance was found regarding age, presence of children, education level, monthly income, economic burden, stage of breast cancer diagnosis, breast surgery, use of painkillers, presence of other diseases, menopausal symptoms, and fatigue-related education.

### Differences in main variables according to return to work

As presented in Table 2, significant associations were found for the following variables. The mean score for subjective fatigue was lower among employed BCS (88.94±34.67) than among those who were not currently employed (102.48±39.84), implying that higher levels of subjective fatigue might impede RTW (t=2.15, *p*=.033). A similar result was found for physical fatigability, as seen by lower levels in participants who had returned to work than in those who had not (23.92±9.62 vs. 28.14±11.34; t=2.19, *p*=.031).

The quality of sleep was better among participants who had returned to work (7.23±3.57) compared to those who had not (8.57±4.11), implying that higher levels of quality of sleep score might impede RTW (t=2.02, *p*=.046). Compared to participants who had returned to work, those who were not currently employed were more likely to have sleep quality scores >8.5, indicating a sleep disorder (t=2.02, *p*=.046). Among the subareas of cancer adaptation, anxious preoccupation showed a statistically significant result (t=3.47, *p*=.001), with higher scores seen in participants who were not employed (23.33±4.54) than in those who had returned to work (20.66±4.33). The mean score for quality of working life was lower among participants who were not currently employed (39.68±21.51) compared to those who had returned to work (55.93±16.23), suggesting that lower levels of quality of working life might impede RTW (t=4.81, *p*<.001).

### Factors associated with return to work

As presented in Table 3, the main variables that demonstrated statistical significance—marital status, time since breast cancer diagnosis, chemo/radiation/target therapy status, fatigue, quality of sleep, mental adjustment, and quality of working life—were included in the multiple logistic regression analysis, as well as cognitive function, all of which are important factors related to workplace function. Marital status, time since breast cancer diagnosis, anxious preoccupation, and quality of working life were found to be predictors of RTW. The explanatory power of this model was 34.0%.

Single participants had a significantly higher likelihood of RTW than those who were married, divorced, separated, or widowed (OR, 3.34; *p*=.027). Participants diagnosed with breast cancer between 2 and 5 years ago had a significantly higher likelihood of RTW than those diagnosed with breast cancer less than 2 years before (OR, 2.85; *p*=.028). As the score for anxious preoccupation increased by 1 point, the likelihood of RTW became lower (OR, 0.89; *p*=.021), and 1-point increases in quality of working life were associated with a higher likelihood of RTW (OR, 1.04; *p*=.010).

## Discussion

The most common reasons given by our BCS participants for change in work status align with those reported in prior research—namely, marital status [29-31], time since breast cancer diagnosis [29,30], anxiety [10,30], and quality of working life [4,31-33]. The aforementioned variables were found to be significant risk factors for one another and were found to be closely related to unemployment/job anxiety in prior research [34]. Consistent with previous research on RTW in BCS [29-31], most participants in this study were young (<50 years old) and married with children. Married BCS, including those who were divorced, separated, and widowed frequently encountered greater difficulties in RTW compared to their single counterparts. Married BCS may have caregiving and household responsibilities that can make it more challenging to manage both work and daily life at home while recovering [35,36]. Married BCS may face greater emotional and psychological burdens related to their roles within the family, and their increased responsibilities can exacerbate feelings of stress about RTW, which can be compounded by the ongoing physical challenges of cancer recovery [37]. In terms of clinical characteristics, most of the BCS in this study were in the early stages of cancer survivorship. In this study, RTW was more frequent 2 years after breast cancer diagnosis and when treatments were complete or not received, reinforcing the evidence from previous research [5,38]. There are challenges in balancing work life and therapy during the acute treatment period, which includes surgery, chemotherapy, radiation therapy, and targeted therapy. Prior research indicates that a more advanced disease stage and extensive surgery can reduce RTW, especially within 3 years of diagnosis [10]. During the treatment phase, within 2 years of a breast cancer diagnosis, BCS may face obstacles in physical recovery, psychological adjustment, and fatigue, all of which can restrict their RTW [37,38]. Thus, to facilitate RTW for BCS, it is essential to provide comprehensive support and counseling to address the physical-psychological challenges with fatigue associated with caregiving or household responsibilities [37].

Regarding mental adjustment to cancer, this study identified high scores of helplessness-hopelessness, anxious preoccupation, and fatalism. Previous research has highlighted the importance of finding individual coping mechanisms [2,15], and the findings from this study can help us better understand the mindset of Korean BCS to tailor more effective RTW interventions. In particular, traditional health beliefs such as fatalism about cancer diagnosis are deeply entrenched among women in many Asian countries, leading to negative mental health outcomes and feelings of helplessness [16]. A high prevalence of depression among Korean BCS has been reported [32], and negative psychosocial factors such as anxiety, anxious preoccupation, fatalism, and depression are associated with fatigue, which can hinder RTW [3,39,40]. This study found that BCS who returned to work had lower levels of anxious preoccupation compared to those who did not. The findings support previous research indicating that BCS who did not return to work experience higher levels of depression, anxiety, and distress [16]. Therefore, returning to work may be associated with improved mental health factors for cancer survivors. To establish causality, however, further research is needed to explore a broader range of psychological factors and intervention strategies.

Quality of work life in our study was lower than that reported in a previous study of BCS in Europe [41]. The moderate quality of working life observed among our participants, along with a lower RTW rate, suggests significant room for improvement in RTW conditions in Korea. In our research, quality of working life emerged as a significant predictor of RTW. BCS who reported a higher quality of working life were more likely to successfully return to and sustain their employment. Consistent with earlier research, our findings highlight the critical role of accessible physical, social, emotional, and informational support [42-44].

Our finding of medium levels of fatigue is consistent with previous research in Western BCS populations [3,9]. Previous Korean research has found fatigue and fitness to be the most frequent physical difficulties impeding RTW among BCS [10]. In comparison to a recent study on BCS with moderate or greater fatigue [34], this study also found low levels of total physical activity. This is particularly noteworthy considering that more than half of the participants were within 2 years of their diagnosis, and physical activity for CRF management and planning for RTW can be helpful after active treatment is completed. Indeed, the variable that showed the most prominent between-group difference in RTW was fatigue. This study showed that both subjective fatigue and physical fatigability were associated with RTW, confirming the findings of previous studies that demonstrated associations between CRF and RTW [2,10,31]. Additionally, given the established positive correlation between exercise and RTW, we recommend providing educational support for regular exercise management and fatigue monitoring at all stages of survivorship to facilitate RTW for BCS [10].

Furthermore, sleep is an important factor for cancer survivors, as sleep disturbances have been found to significantly increase healthcare expenditures and absenteeism in the United States [45]. Previous studies have indicated that insomnia is twice as prevalent among BCS, with concurrent and heightened symptoms of sleep disturbance linked to greater cancer-related uncertainty and fatigue, particularly in younger BCS (<50 years) [9]. As our study population also exhibited borderline poor sleep quality, efforts to emphasize the importance of sleep are critical in interventions and support programs for Korean BCS [2].

In order to increase RTW, some Western countries provide a wide range of detailed information through online sources [46]. Improving the availability of RTW information online and in the workplace for Korean BCS may help normalize RTW and provide opportunities for colleagues, supervisors, and employers to understand and support their colleagues who are cancer survivors [42]. We advocate for a dual emphasis on both physical and psychosocial rehabilitation—including physical therapy, exercise programs for fatigue management, psychoeducation, and sleep support—in RTW programs, alongside vocational resources such as occupational counseling and the involvement of vocational experts in creating tailored RTW plans [2,46]. In Korea, the development and implementation of such multidisciplinary interventions will necessitate comprehensive coordination among oncologists, clinical psychologists, social workers, occupational experts, and workplace managers and employers [46]. As nurses are at the frontline of treatment for BCS, they are well-positioned to assess issues like fatigue, sleep disturbances, and anxiety, and to utilize study findings to educate and support RTW, particularly in roles as liaisons and resource providers.

The strengths of this study included the analysis of understudied RTW parameters and its focus on an understudied population of BCS regarding RTW. A limitation of this study was the inability to infer causality due to the cross-sectional design. More longitudinal and intervention-based studies are needed to examine RTW in Korean BCS. Additionally, the small sample size of this study may limit the generalizability of its findings. Future studies on RTW would benefit from a more detailed focus on specific types of employment, including professional and managerial roles, positions requiring significant physical effort, self-employment, and jobs that involve night shifts or temporary work.

In conclusion, this study of Korean BCS found both subjective fatigue and physical fatigability to be strongly negatively associated with RTW, while high-quality sleep appeared to have a positive association. Anxious preoccupation also showed a significant relationship with RTW, highlighting the importance of including a variety of psychological factors in future RTW analyses. Previous studies in Korea have primarily focused on the clinical aspects of recovery and the prevention of cancer recurrence. However, there is a growing need to shift attention towards cancer survivorship. Future initiatives should aim to enhance physical and psychosocial support resources and develop RTW interventions tailored to Korean BCS. These interventions should focus on alleviating CRF, reducing anxious preoccupation, and improving the quality of working life.

## Figures and Tables

**Table 1.** Return to work according to general characteristics (N=135)

Characteristic	Categories	Total	Working (n=77)	Not currently working (n=58)	χ^2^ or F	*p*	Scheffé test
Age (year)	20–39	36 (26.7)	18 (50.0)	18 (50.0)	2.86	.414	
	40–49	65 (48.1)	38 (58.5)	27 (41.5)			
	50–59	23 (17.0)	16 (69.6)	7 (30.4)			
	60–69	11 (8.1)	5 (45.5)	6 (54.5)			
Marital status	Married/divorced/separated/widowed	106 (78.5)	55 (51.9)	51 (48.1)	5.34	.021	
	Single	29 (21.5)	22 (75.9)	7 (24.1)			
Children	Yes	91 (67.4)	29 (65.9)	15 (34.1)	2.10	.148	
	None	44 (32.6)	48 (52.7)	43 (47.3)			
Education level	Up to high school	39 (28.9)	23 (59.0)	16 (41.0)	0.08	.772	
	≥College or higher	96 (71.1)	54 (56.4)	42 (43.6)			
Monthly income (KRW)	<3 million	35 (25.9)	19 (54.3)	16 (45.7)	2.37	.306	
	3–5 million	58 (43.0)	30 (51.7)	28 (48.3)			
	>5 million	42 (31.1)	28 (66.7)	14 (33.3)			
Economic burden	Not at all/not too burdensome	33 (24.4)	24 (72.7)	9 (27.3)	4.64	.098	
	Average	40 (29.6)	22 (55.0)	18 (45.0)			
	A little/very burdensome	62 (45.9)	31 (50.0)	31 (50.0)			
Time since breast cancer diagnosis (year)^†^	Up to 2^a^	84 (62.2.)	40 (47.6)	44 (52.4)	8.05	.018	a<b
	2–5^b^	44 (32.6)	32 (72.7)	12 (27.3)			
	>5^c^	7 (5.2)	5 (71.4)	2 (28.6)			
Breast cancer diagnosis stage	Stage 0–2	117 (86.7)	69 (59.0)	48 (41.0)	1.64	.504	
	Stage 3–4	15 (11.1)	7 (46.7)	8 (53.3)			
	Unknown	3 (2.2)	1 (33.3)	2 (66.7)			
Breast surgery	Yes	118 (87.4)	67 (56.8)	51 (43.2)	1.53	.466	
	No	16 (11.9)	10 (62.5)	6 (37.5)			
	Unknown	1 (0.7)	0 (0)	1 (100)			
Chemo/radiation/target therapy^†^	Ongoing^a^	33 (24.5)	11 (33.3)	22 (66.7)	10.55	.004	a<b, c
	Completed^b^	87 (64.4)	55 (63.2)	32 (36.8)			
	None^c^	21 (11.1)	11 (73.3)	4 (26.7)			
Use of painkillers	Yes	37 (27.4)	19 (51.4)	18 (48.6)	0.90	.638	
	No	89 (65.9)	52 (58.4)	37 (41.6)			
	Not applicable	9 (6.7)	6 (66.7)	3 (33.3)			
Other diseases	Yes	33 (24.4)	20 (60.6)	13 (39.4)	0.23	.634	
	None	102 (75.6)	57 (55.9)	45 (44.1)			
Menopausal symptoms and discomfort	Yes	82 (60.7)	46 (59.7)	36 (62.1)	2.33	.370	
	No	53 (39.3)	31 (40.3)	22 (37.9)			
Fatigue-related education^†^	Yes	11 (8.1)	70 (56.5)	54 (43.5)	0.21	.645	
	No	124 (91.9)	7 (63.6)	4 (36.4)			

KRW: Korean won (1 million KRW=roughly 900 US dollars).

† Post-hoc analysis.

**Table 2.** Differences in main variables according to return to work (N=135)

Variable	Possible range	Study range	Mean±SD			t (*p*)
			Total	Working (n=77)	Not currently working (n=58)	
Subjective fatigue	0–190	14–172	94.88±37.47	88.94±34.67	102.48±39.84	2.15 (.033)
Activity-related fatigue						
Physical fatigability	0–50	1–48	25.73±10.56	23.92±9.62	28.14±11.34	2.19 (.031)
Mental fatigability	0–50	0–46	21.87±11.15	20.23±10.57	24.02±11.63	1.88 (.062)
Quality of sleep	0–21	1–17	7.81±3.85	7.23±3.57	8.57±4.11	2.02 (.046)
Adjustment to cancer						
Helplessness-Hopelessness	8–32	8–30	15.16±4.80	14.64±4.99	15.84±4.49	1.45 (.149)
Anxious preoccupation	8–32	9–31	21.81±4.60	20.66±4.33	23.33±4.54	3.45 (.001)
Fighting spirit	4–16	7–16	11.78±1.97	11.77±1.84	11.79±2.13	0.08 (.938)
Cognitive avoidance	4–16	4–16	10.56±2.59	10.52±2.46	10.60±2.78	0.19 (.853)
Fatalism	5–20	6–20	14.31±2.59	14.18±2.44	14.48±2.79	0.67 (.506)
Quality of working life	0–100	2–89	48.95±20.28	55.93±16.23	39.68±21.51	4.81 (<.001)

**Table 3.** Factors influencing return to work (N=135)

Factor	Categories	B	SE	*p*	OR (95% CI)
Marital status	Single	1.21	0.55	.027	3.34 (1.15–9.72)
Time since breast cancer diagnosis (year)	2–5	1.05	0.48	.028	2.85 (1.12–7.26)
	>5	0.45	0.98	.643	1.57 (0.23–10.62)
Chemo/radiation/target therapy	Completed	1.00	0.52	.055	2.73 (0.98–7.58)
	None	1.52	0.80	.059	4.56 (0.94–22.04)
Fatigue factor	Subjective fatigue	–0.01	0.01	.315	0.99 (0.98–1.01)
	Physical fatigability	0.04	0.04	.315	1.04 (0.96–1.12)
	Mental fatigability	–0.04	0.03	.251	0.96 (0.90–1.03)
Sleep factor	Quality of sleep	–0.08	0.06	.190	0.92 (0.81–1.04)
Adjustment to cancer factor	Anxious preoccupation	–0.12	0.05	.021	0.89 (0.80–0.98)
Psychosocial factors	Quality of working life	0.04	0.02	.010	1.04 (1.01–1.08)
R^2^=0.34, Hosmer & Lemeshow test χ^2^=3.56, *p*=.895					

OR: odds ratio; CI: confidence interval.
